# Editorial

**Published:** 1896-05

**Authors:** 


### EDITORIAL.

Already the signs of the changes on the college-chart are looming up over the horizon of the future educational forces in America, and, with three new schools in the band having a curriculum of three years of six months each, obligatory on the students entering the sessions of 1896-97, the most sanguine and hopeful friend of higher veterinary education will rejoice beyond measure at such an outlook. With another school about to undergo changes through the loss of two of its most valuable instructors, because they believe the time for a longer curriculum has arrived, and their college does not, means another victory for the cause that is little less in importance than the step taken by the other schools. This will leave but three schools on the list of two-term requirements, and the growth of State boards of examiners and the demand for more broadly educated men will soon decide the future of these schools and win victories for this grand cause, that will surely come, whether reluctantly conceded or by the force of circumstances that shall limit and circumscribe their sphere to a narrow field, wholly unacceptable to those who have long directed these institutions. Let the good work go on!

---

President Haines, of the American Society for the Prevention of Cruelty to Animals, is out in a leaflet relative to the operation of docking. After a brief review of the anatomy of the parts, especially the sensory nerve-supply, the common method of operating—with a twitch, one foot tied up, the docking knife, and cautery—is described, its painful character under such methods is forcibly alluded to, and opinions condemning the operation as one of fashion and cruelty are quoted from veterinarians Prof. George Fleming, Professor Pritchard, Professor Zuill, Professor Liautard, Doctors S. K. Johnson, L. McLean, and John. W. Gadsden. We join with President Haines in wishing that the operation, as one of fashion, and especially in the manner described, may be forever abandoned. The operation as performed to-day is largely done by grooms, horsedealers, and others, but, as well said by Professor Haines, with the consent and acquiescence of owners; and we would say even more, that we have known active personages in the Society of which President Haines is the honored head to decline to have horses docked, and in the same breath decline to purchase any save those with docked tails.

---

ANOTHER IN THE FOLD. Just as we are about to go to press with the May number comes the welcome news that the Chicago Veterinary College will, with its opening session of October, 1896, enter the roll of schools with a curricula of three years of six months each. This school, the oldest of all the Western schools, and with a very large alumni scattered over the entire West, enters the number with a prestige and strength back of it unlike any of the other three-year schools west of the Allegheny Mountains, and we are sure it will prove a tower of strength and support in this fresh determination to be among the foremost schools of our country. Well may the profession rejoice; well may the United States Association members feel proud of this accession to the ranks, and all honor to the Association of Faculties, whose strong advocates and supporters have so well and conservatively, without bond or fetter, drawn every school to its recognition, and won support in its efforts in fields where the mere suggestion of legal ties and bonds would have driven away many of those who now worship at its altar.

---

THE JOURNAL regrets exceedingly to learn that Professors Brenton and McEachran have severed their connection with the Detroit Veterinary College, and specially so for the reasons assigned. Higher veterinary education has a sincere advocate in Professor Brenton, and the courage of his convictions is more than demonstrated by his refusal to continue longer with a school whose course did not command his earnest approval and hearty support.

---

THE Committee on Army Legislation, undismayed by unfavorable reports from the Quartermaster-General's office, continued to press the present bill before the House Military Committee, and, having eliminated the clause that confined future appointments to graduates of veterinary colleges of the United States, have been enabled to secure, through the kindly aid of Chairman Hull, the favorable approval of the bill, and the same placed on the calendar of the lower House. The Senate committee will await the action of the House in dealing with the bill, and its passage through the latter will place it in a strong position for consideration in the upper branch, which is much to be desired from all points of view; for in the event of failure at this session it gives strong prestige for the bill in the next session of Congress, and all should aid its advancement in every way they can.

---

MARRIED. At Philadelphia, April 18th, by Friends' ceremony, Dr. Henry W. Turner, of Lahaska, graduate of the Veterinary Department University of Pennsylvania, and Miss May Atkinson, of Solebury, Pa.

DIED. At St. Louis, Mo., on March 19th, Dr. Charles Hesse, an old and well-known practitioner in that city.

